# Assessment of Serum Concentrations of Adropin, Afamin, and Neudesin in Children with Type 1 Diabetes

**DOI:** 10.1155/2019/6128410

**Published:** 2019-10-24

**Authors:** Agnieszka Polkowska, Izabela Elżbieta Pasierowska, Marta Pasławska, Elżbieta Pawluczuk, Artur Bossowski

**Affiliations:** Department of Pediatrics, Endocrinology, Diabetology with Subdivision of Cardiology, Medical University of Białystok, Białystok, Poland

## Abstract

**Introduction:**

The increasing knowledge of adropin, afamin, and neudesin and the regulation of glucose metabolism and insulin resistance allows for the assessment of the differences in their concentrations between the groups with varied duration of diabetes mellitus (DM).

**Aim of the Study:**

Assessment of serum levels of adropin, afamin, and neudesin in children with type 1 diabetes, with respect to the disease duration.

**Materials and Methods:**

The study consisted of 138 patients aged 5–18 years (M 40.58%). Children with type 1 diabetes (*n* = 68) were compared to the control group (*n* = 70). The diabetic group was divided into 4 subgroups: (I) newly diagnosed patients, after an episode of ketoacidosis (*n* = 14), (II) duration no longer than 5 years (*n* = 18), (III) 5 to 10 years (*n* = 27), and (IV) longer than 10 years (*n* = 9). Serum concentrations of adropin, afamin, and neudesin were assessed and compared between the groups of patients. The criterion for statistical significance was *p* < 0.05.

**Results:**

The concentrations of adropin and afamin across all subgroups were lower than that in the control group, while neudesin levels were higher in diabetic patients compared to the control group. The differences were statistically significant.

**Conclusions:**

Adropin, afamin, and neudesin may play a major role in the regulation of glucose metabolism and have a significant potential as novel biomarkers to predict future metabolic disorders. However, further multicentre studies on a larger cohort of patients are necessary to specify the role of these substances in the course and treatment of type 1 diabetes.

## 1. Introduction

The increasing knowledge of the regulatory peptides allows for the assessment of their role in linking the process of food intake, nutritional status, and body growth, largely through the regulation of glucose metabolism and insulin resistance [1]. One of the regulatory peptides is adropin, a product of the energy homeostasis associated (Enho) gene, which has been identified as a potent regulatory hormone responsible for the maintenance of glucose tolerance and insulin sensitivity in mice [2, 3]. This peptide is mainly synthetized in the liver and brain, and its prime function is to regulate carbohydrate, lipid, and protein metabolism via moderating glucose-mediated insulin release [4]. The expression of adropin in the central nervous system suggests that this adipokine may play a role as a neuropeptide [3]. Moreover, it is possible that adropin has autocrine/paracrine roles in peripheral tissues [3]. In addition, adropin exerts an endothelial protective effect and might increase nitric oxide (NO) release, activate endothelial nitric oxide synthase (eNOS), activate vascular endothelial growth factor receptor 2 (VEGFR2), and modulate neovascularization [5].

Another peptide is afamin, a member of the human albumin gene family which also includes albumin, vitamin D-binding protein, and fetoprotein [6]. This glycoprotein is mainly expressed in the liver and secreted into the blood stream [7]. It is suggested that afamin could be involved in the regulation of α-tocopherol uptake and transport at the blood-brain barrier [8]. This protein also plays a role in antiapoptotic cellular processes related to oxidative stress and is associated with insulin resistance and other features of the metabolic syndrome [9].

The third substance assessed in our paper is neudesin. Mouse neudesin mRNA expression is detected in various tissues including the brain, heart, lung, and kidney [10]. In the examination of the mouse embryonic cerebral cortex and cultured mouse neural precursor cells, neudesin significantly promotes neuronal differentiation [11]. In addition, in some studies using rodent models, neudesin knockout (KO) mice were protected from insulin resistance induced by high-fat diet (HFD) [12]. This may suggest that neudesin is an important factor in the central regulation of the energy metabolism.

Most studies assessing the relationship of adropin, afamin, and neudesin with glucose metabolism provide data obtained from research conducted on animal models, adults with type 2 diabetes, and women with gestational diabetes. There are only few studies concerning these relationships in children.

## 2. Aim of the Study

 The study objective was to evaluate the concentrations of the selected peptides in blood serum of children with type 1 diabetes, taking into account the disease duration.

## 3. Materials and Methods

The study population consisted of 138 children aged 5–18 years (male 40.58%, M/F = 0.68), diabetic patients of Diabetology Outpatient Clinic and Department of Pediatrics, Endocrinology, Diabetology with Subdivision of Cardiology, Medical University of Bialystok. The population included a group of 68 children with type 1 diabetes (diagnosed by ISPAD criteria) as compared to the control group of 70 healthy children with a negative history of inflammatory, autoimmune diseases, or cancer. The diabetic group was divided into 4 subgroups regarding the duration of type 1 diabetes: (I) newly diagnosed patients, after an episode of ketoacidosis (*n* = 14), (II) duration no longer than 5 years (*n* = 18), (III) 5 to 10 years (*n* = 27), and (IV) longer than 10 years (*n* = 9). Statistical analysis was based on anthropometric parameters such as age, height, and weight and laboratory tests (HbA1c) using standard methods. We analyzed medical records of the patients with type 1 diabetes (method of insulin therapy and other diseases). Moreover, we used ELISA to measure serum concentrations of adropin, afamin, and neudesin in the patients. Statistical analysis was performed using Statistica 13.0. The nonparametric *U* Mann–Whitney test was applied to compare quantitative variables without normal distribution.

## 4. Results

**Figure 1:**
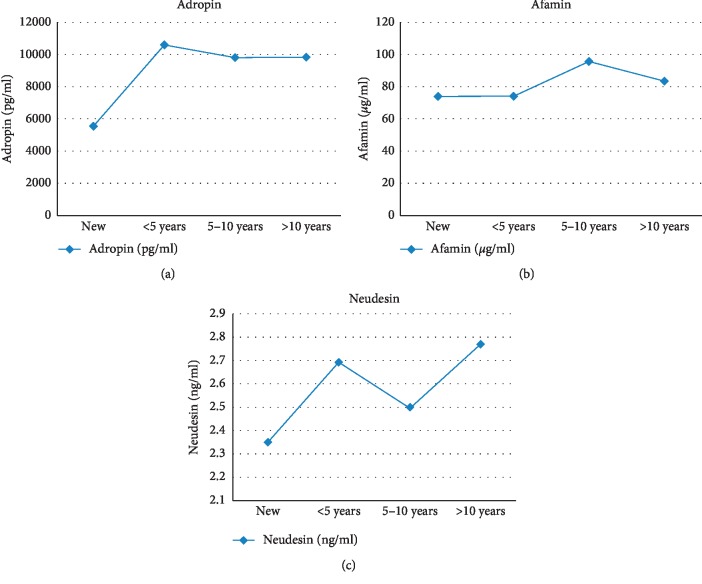
The concentration of (a) adropin, (b) afamin, and (c) neudesin in the serum of children with type 1 diabetes, with respect to disease duration.

The mean levels of adropin and afamin in all subgroups were lower than in the control group (Table 1). The mean level of adropin in children with type 1 DM varied from min. 5558.45 pg/ml (subgroup I) to max. 10579.00 pg/ml (subgroup II) vs. control group 13035.18 pg/ml. The mean level of afamin varied from min. 74.09 *μ*g/ml (subgroup I) to max. 95.44 *μ*g/ml (subgroup III) vs. control group 116.91 *μ*g/ml. The mean levels of adropin and afamin were found to fluctuate during the course of the disease, whereas the mean level of neudesin remained stable (Figures 1(a)–1(c)). The mean levels of adropin and afamin were lower in newly diagnosed children than in those with the longest disease duration (over 10 years). The level of neudesin was stable over the disease progression (2.35–2.77 ng/ml) and higher than in the control group 1.95 ng/ml.

**Figure 2:**
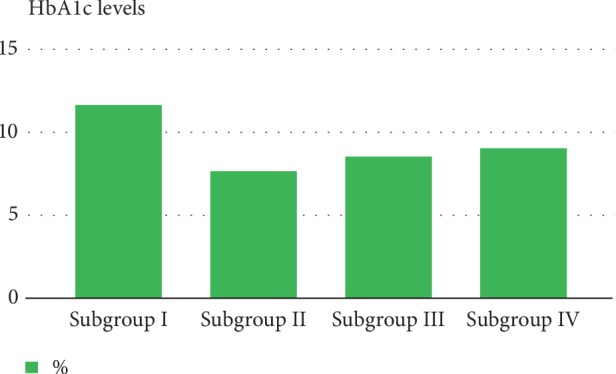
Mean HbA1c values in subgroups with type 1 diabetes. Statistical significance between subgroup I and subgroup II is *p* < 0.00001. Statistical significance between subgroup I and subgroup III is *p* < 0.00002. Statistical significance between subgroup I and subgroup IV is *p* < 0.02.

The mean glycated hemoglobin (HbA1c) level was the highest in subgroup I (11.69%) as compared to subgroups II, III, and IV due to the fact that it contained recently diagnosed cases of type 1 diabetes. The second highest mean level of Hb1Ac was observed in the group of children with the longest disease duration (9.06%). The mean HbA1c among children in subgroups II and III was comparable (7.67% vs. 8.58%) (Figure 2).

Patients treated with continuous subcutaneous insulin infusion (CSII) had higher mean levels of all the peptides and lower mean HbA1c levels than children treated with multiple daily injections of insulin (MDI) (*p*=0.000001).

The analysis of anthropometric parameters revealed that the groups did not differ statistically significantly in terms of BMI. The newly diagnosed children where statistically younger (*p*=0.0005) and had lower body weight (*p*=0.00542) and height (*p*=0.0398) (Table 2).

Moreover, we noted that diabetic patients with coexistent diseases were statistically younger (*p*=0.007176) and had a higher BMI (*p*=0.033627) than diabetic children without any other disease.

Furthermore, our study showed a statistically significant correlation between BMI and the levels of adropin in subgroup II, as well as between BMI and the levels of neudesin in children with the longest disease duration. In addition, we found a statistically significant correlation of adropin and afamin with age, weight, and height in children with up to 5 years' of lasting diabetes.

## 5. Discussion

Over the past few years, scientists have discovered new regulatory peptides involved in the regulation of carbohydrate metabolism. Numerous studies concerning different fields of medicine are still revealing their novel properties. A thorough understanding of the mechanisms of these peptides and factors which influence their release may provide new possibilities in the diagnosis and treatment of metabolic disorders, including type 1 diabetes. Most papers assessing the relationship of adropin, afamin, and neudesin with glucose metabolism provide research data from animal models, adults with type 2 diabetes, and women with gestational diabetes. There are only few studies concerning these relationships in children but not in those with type 1 diabetes. In the current study, we evaluated levels of these regulatory peptides in children with type 1 diabetes, with respect to the disease duration.

The results of our analysis showed that the mean levels of adropin and afamin were statistically lower in children with type 1 diabetes as compared to the control group, whereas mean neudesin concentration was statistically higher in diabetic patients. Similar correlations were observed by Zang et al. [13] and Wu et al. [14], who found lower serum adropin levels in type 2 diabetic patients than that in nondiabetic children. However, in other studies, adropin levels were observed to be significantly higher in patients with type 2 diabetes compared to healthy controls [15]. Reports by Beigi et al. [16] indicated a significant difference in adropin concentrations between women with gestational diabetes and healthy pregnant females, with adropin levels being lower in the gestational diabetes group. On the contrary, in a study conducted by Köninger et al., patients who developed gestational diabetes had significantly higher afamin concentrations during the first trimester than those without diabetes in their ongoing pregnancy [9].

Our analysis showed that higher levels of regulatory substances were accompanied by a higher body mass index (BMI). A significant positive correlation between afamin levels and BMI was observed in a study by Paragh et al. [17]. However, Kratochvilova et al. found negative relationships between serum levels of neudesin and BMI [18]. In addition, reports by Altincik and Sayin [19] and Sayın et al. [20] indicated that serum adropin levels were significantly lower in obese patients as compared to children with normal weight. The results of research in obese adults showed that adropin levels were also lower in these individuals [21]. Moreover, in a study conducted by Chang et al., a negative correlation was observed between plasma adropin concentrations and waist-to-hip ratios (WHR) and lower body fat percentage by mass [22].

The results of the present analysis showed a negative correlation between serum concentrations of regulatory peptides and level of glycated hemoglobin (HbA1c). Similar relationships were observed in a study by Kratochvilova et al., conducted in a group of obese adults with type 2 diabetes [18]. However, in the research performed by Dąbrowski et al., serum adropin levels positively correlated with HbA1c among women with gestational diabetes [23].

In our study, patients treated with continuous subcutaneous insulin infusion (CSII) had significantly lower mean HbA1c levels than children treated with multiple daily injections (MDI) of insulin. Similar correlations were observed in studies conducted by Beck et al. [24] and Ruiz-de-Adana et al. [25], in which glycemic control was improved by initiation of CSII in adults with type 1 diabetes. Reports by Patton et al. also indicated an association of CSII with reduced levels of HbA1c in children newly diagnosed with type 1 diabetes [26]. Similar relationships were confirmed by Rys et al. among pregnant women with type 1 diabetes, where CSII compared to MDI therapy resulted in better glycemic control during pregnancy, and CSII therapy was associated with lower insulin requirements [27].

In the present analysis, the levels of the regulatory peptides were higher in children with coexistent diseases, such as autoimmune thyroid diseases, dyslipidemia, celiac disease, obesity, metabolic disorders, and hypertension. In a study by Köninger et al., afamin concentrations were found to be significantly increased in patients with polycystic ovary syndrome (PCOS) in comparison with controls [28]. Kronenberg et al. [29] and Seeber et al. [30] reported a significant association between afamin plasma concentrations and individual anthropometric and metabolic risk factors, being relevant for the development of metabolic syndrome. It is known that women suffering from PCOS frequently develop metabolic complications. Thus, afamin can serve as a prognostic factor for the future development of metabolic syndrome in young individuals, especially women with insulin resistance. However, an inverse association was also observed between plasma adropin levels and metabolic syndrome, nonalcoholic fatty liver disease, and PCOS [20, 31, 32].

Insulin resistance is an important factor that affects the clinical course and metabolic control in patients with both type 1 and type 2 diabetes. In children with type 1 diabetes, insulin resistance is higher in comparison to their healthy peers [33]. Several animal studies have been performed concerning the role of adropin and afamin in glucose and insulin homeostasis. Adropin has been identified as a potent regulatory hormone responsible for the maintenance of glucose tolerance and insulin sensitivity in murine models [2, 3]. The available data indicate that adropin treatment in mice enhances glucose tolerance and improves insulin resistance [34]. In a rat model of type 2 diabetes, treatment with adropin could increase insulin sensitivity and reduce blood glucose level and insulin resistance [35]. In a study performed by Ohta et al., in wild-type mice, glucose tolerance was impaired and insulin sensitivity was aggravated by high-fat diet (HFD), whereas in neudesin knockout (KO) mice, both effects were improved [12]. It may suggest that neudesin KO mice were protected from insulin resistance induced by HFD [12]. As already mentioned, diabetic children have higher insulin resistance than their healthy peers [33]. This may explain why patients with type 1 diabetes have lower adropin levels and higher neudesin concentrations than nondiabetic children.

To sum up, it should be emphasized that regulatory peptides can modulate insulin sensitivity both in healthy individuals and people with autoimmune diseases such as type 1 diabetes. Their serum concentrations not only affect glucose utilization [34] but also exert long-term effects on the whole energy homeostasis [36]. As mentioned previously, the differences in the levels of the active substances assessed in our study depend on BMI, metabolic control of diabetes, and the disease duration. Patients with normal endocrine function of the pancreas have, respectively, higher levels of adropin and afamin and lower level of neudesin as compared to individuals suffering from type 1 diabetes. Moreover, the levels of these peptides vary between patients with preserved residual pancreatic secretory function (in remission) and those with long duration of type 1 diabetes. In our analysis, we observed an increase in the mean levels of adropin and afamin correlated to longer duration of the disease, whereas the mean level of neudesin remained stable.

Based on the data obtained from animal studies, adults with type 2 diabetes, and women with gestational diabetes, it seems that adropin, afamin, and neudesin play a major role in the regulation of glucose metabolism and insulin sensitivity. This regulatory peptides have a significant potential as novel biomarkers to predict future metabolic disorders. However, further multicentre studies on a larger cohort of patients are necessary to specify the role of these substances in the course and treatment of type 1 diabetes.

## 6. Conclusions

1. The levels of adropin and afamin may be associated with the time of current type 1 diabetes and may change during its course. There is a need for more research connected with this subject.
2. BMI and HbA1c can affect the levels of the regulatory peptides in the body.

## Figures and Tables

**Table 1:** Mean concentrations of adropin, afamin, and neudesin in blood serum in the study groups with mean values and SD.

Hormone	Subgroup I	Subgroup II	Subgroup III	Subgroup IV	Control group	*p*, ^*∗*^*p*, ^*∗∗*^*p*, ^*∗∗∗*^*p*
Adropin (pq/ml)	5558.45 ± 2707.61	10579.00 ± 9632.96	9793.96 ± 6348.57	9843.15 ± 6047.97	13035.18 ± 6946.50	*p*=0.000164, ^*∗*^*p*=0.240428, ^*∗∗*^*p*=0.028781, ^*∗∗∗*^*p*=0.245476

Afamin (*μ*g/ml)	74.09 ± 29.29	74.10 ± 17.73	95.44 ± 29.81	83.27 ± 18.63	116.91 ± 25.55	*p* < 0.0001, ^*∗*^*p* < 0.0001, ^*∗∗*^*p*=0.000357, ^*∗∗∗*^*p*=0.001134

Neudesin (ng/ml)	2.35 ± 0.47	2.69 ± 0.77	2.50 ± 0.70	2.77 ± 0.63	1.95 ± 0.69	*p*=0.01790, ^*∗*^*p*=0.021382, ^*∗∗*^*p*=0.076555, ^*∗∗∗*^*p*=0.000628

*p*: statistical significance between subgroup I and control group; ^*∗*^*p*: statistical significance between subgroup II and control group; ^*∗∗*^*p*: statistical significance between subgroup III and control group; ^*∗∗∗*^*p*: statistical significance between group IV and control group.

**Table 2:** Basic characteristics of the study groups.

Characteristics	Subgroup I	Subgroup II	Subgroup III	Subgroup IV	Control group	*p*, ^*∗*^*p*, ^*∗∗*^*p*, ^*∗∗∗*^*p*
Age (years)	11.03 ± 3.57	11.78 ± 4.85	14.90 ± 2.58	15.93 ± 1.79	14.01 ± 2.64	*p*=0.0005, ^*∗*^*p*=0.011313, ^*∗∗*^*p*=0.116992, ^*∗∗∗*^*p*=0.06502
Height (cm)	148.89 ± 21.88	147.56 ± 25.47	163.67 ± 15.18	167.21 ± 7.85	160.53 ± 18.39	*p*=0.039792, ^*∗*^*p*=0.020999, ^*∗∗*^*p*=0.40669, ^*∗∗∗*^*p*=0.346118
Body mass (kg)	43.06 ± 15.51	44.99 ± 21.39	59.40 ± 15.78	62.16 ± 12.22	60.18 ± 21.27	*p*=0.005423, ^*∗*^*p*=0.01180, ^*∗∗*^*p*=0.854739, ^*∗∗∗*^*p*=0.81048
BMI (kg/m^2^)	18.78 ± 2.95	19.23 ± 3.16	21.86 ± 3.69	22.05 ± 2.67	23.23 ± 9.03	*p*=0.073051, ^*∗*^*p*=0.08676, ^*∗∗*^*p*=0.417367, ^*∗∗∗*^*p*=0.73374

*p*: statistical significance between subgroup I and control group; ^*∗*^*p*: statistical significance between subgroup II and control group; ^*∗∗*^*p*: statistical significance between subgroup III and control group; ^*∗∗∗*^*p*: statistical significance between group IV and control group.

## Data Availability

The data used to support the findings of this study are available from the corresponding author upon request.

## References

[1] Polkowska A., Szczepaniak I., Bossowski A. (2016). Assessment of serum concentrations of ghrelin, obestatin, omentin-1, and apelin in children with type 1 diabetes. *BioMed Research International*.

[2] Kumar K. G., Trevaskis J. L., Lam D. D. (2008). Identification of adropin as a secreted factor linking dietary macronutrient intake with energy homeostasis and lipid metabolism. *Cell Metabolism*.

[3] Ganesh Kumar K., Zhang J., Gao S. (2012). Adropin deficiency is associated with increased adiposity and insulin resistance. *Obesity*.

[4] Aydin S. (2014). Three new players in energy regulation: preptin, adropin and irisin. *Peptides*.

[5] Lovren F., Pan Y., Quan A. (2010). Adropin is a novel regulator of endothelial function. *Circulation*.

[6] Lichenstein H. S., Lyons D. E., Wurfel M. M. (1994). Afamin is a new member of the albumin, alpha-fetoprotein, and vitamin D-binding protein gene family. *Journal of Biological Chemistry*.

[7] Kronenberg F., Dieplinger H. (2015). Afamin is a promising novel marker for metabolic syndrome and related diseases. *Clinical Lipidology*.

[8] Kratzer I., Bernhart E., Wintersperger A. (2009). Afamin is synthesized by cerebrovascular endothelial cells and mediates *α*-tocopherol transport across anin vitromodel of the blood-brain barrier. *Journal of Neurochemistry*.

[9] Köninger A., Mathan A., Mach P. (2018). Is afamin a novel biomarker for gestational diabetes mellitus? A pilot study. *Reproductive Biology and Endocrinology*.

[10] Kimura I., Yoshioka M., Konishi M., Miyake A., Itoh N. (2005). Neudesin, a novel secreted protein with a unique primary structure and neurotrophic activity. *Journal of Neuroscience Research*.

[11] Kimura I., Konishi M., Miyake A., Fujimoto M., Itoh N. (2006). Neudesin, a secreted factor, promotes neural cell proliferation and neuronal differentiation in mouse neural precursor cells. *Journal of Neuroscience Research*.

[12] Ohta H., Konishi M., Kobayashi Y. (2015). Deletion of the neurotrophic factor neudesin prevents diet-induced obesity by increased sympathetic activity. *Scientific Reports*.

[13] Zang H., Jiang F., Cheng X., Xu H., Hu X. (2018). Serum adropin levels are decreased in Chinese type 2 diabetic patients and negatively correlated with body mass index. *Endocrine Journal*.

[14] Wu L., Fang J., Chen L. (2014). Low serum adropin is associated with coronary atherosclerosis in type 2 diabetic and non-diabetic patients. *Clinical Chemistry and Laboratory Medicine*.

[15] Hosseini A., Shanaki M., Emamgholipour S., Nakhjavani M., Razi F., Golmohammadi T. (2016). Elevated serum levels of adropin in patients with type 2 diabetes mellitus and its association with insulin resistance. *Journal of Biology and Today’s World*.

[16] Beigi A., Shirzad N., Nikpour F., Nasli Esfahani E., Emamgholipour S., Bandarian F. (2015). Association between serum adropin levels and gestational diabetes mellitus; a case-control study. *Gynecological Endocrinology*.

[17] Paragh G., Lorincz H., Somodi S., Varga V. E., Harangi M., Seres I. (2017). Serum afamin level is strongly associated to the components of metabolic syndrome in non-diabetic obese patients. *Atherosclerosis*.

[18] Kratochvilova H., Lacinova Z., Klouckova J. (2018). Neudesin, a novel regulator of energy metabolism in obesity and type 2 diabetes mellitus—the effect of acute fasting and endoscopic duodenal-jejunal bypass liner implantation. *Diabetes*.

[19] Altincik A., Sayin O. (2015). Evaluation of the relationship between serum adropin levels and blood pressure in obese children. *Journal of Pediatric Endocrinology and Metabolism*.

[20] Sayın O., Tokgöz Y., Arslan N. (2014). Investigation of adropin and leptin levels in pediatric obesity-related nonalcoholic fatty liver disease. *Journal of Pediatric Endocrinology and Metabolism*.

[21] Butler A. A., Tam C. S., Stanhope K. L. (2012). Low circulating adropin concentrations with obesity and aging correlate with risk factors for metabolic disease and increase after gastric bypass surgery in humans. *The Journal of Clinical Endocrinology & Metabolism*.

[22] Chang J.-B., Chu N.-F., Lin F.-H., Hsu J.-T., Chen P.-Y. (2018). Relationship between plasma adropin levels and body composition and lipid characteristics amongst young adolescents in Taiwan. *Obesity Research & Clinical Practice*.

[23] Dąbrowski F. A., Jarmużek P., Gondek A., Cudnoch-Jędrzejewska A., Bomba-Opoń D., Wielgoś M. (2016). First and third trimester serum concentrations of adropin and copeptin in gestational diabetes mellitus and normal pregnancy. *Ginekologia Polska*.

[24] Beck R. W., Riddlesworth T. D., Ruedy K. J. (2017). Effect of initiating use of an insulin pump in adults with type 1 diabetes using multiple daily insulin injections and continuous glucose monitoring (DIAMOND): a multicentre, randomised controlled trial. *The Lancet Diabetes & Endocrinology*.

[25] Ruiz-de-Adana M. S., Dominguez-Lopez M.-E., Gonzalez-Molero I. (2016). Comparison between a multiple daily insulin injection regimen (basal once-daily glargine plus mealtime lispro) and continuous subcutaneous insulin infusion (lispro) using continuous glucose monitoring in metabolically optimized type 1 diabetes patients: a randomized open-labelled parallel study. *Medicina Clínica*.

[26] Patton S. R., Noser A. E., Youngkin E. M., Majidi S., Clements M. A. (2019). Early initiation of diabetes devices relates to improved glycemic control in children with recent-onset type 1 diabetes mellitus. *Diabetes Technology & Therapeutics*.

[27] Rys P. M., Ludwig-Slomczynska A. H., Cyganek K., Malecki M. T. (2018). Continuous subcutaneous insulin infusion vs multiple daily injections in pregnant women with type 1 diabetes mellitus: a systematic review and meta-analysis of randomised controlled trials and observational studies. *European Journal of Endocrinology*.

[28] Köninger A., Edimiris P., Koch L. (2014). Serum concentrations of afamin are elevated in patients with polycystic ovary syndrome. *Endocrine Connections*.

[29] Kronenberg F., Kollerits B., Kiechl S. (2014). Plasma concentrations of afamin are associated with the prevalence and development of metabolic syndrome. *Circulation: Cardiovascular Genetics*.

[30] Seeber B., Morandell E., Lunger F., Wildt L., Dieplinger H. (2014). Afamin serum concentrations are associated with insulin resistance and metabolic syndrome in polycystic ovary syndrome. *Reproductive Biology and Endocrinology*.

[31] Yosaee S., Khodadost M., Esteghamati A. (2017). Metabolic syndrome patients have lower levels of adropin when compared with healthy overweight/obese and lean subjects. *American Journal of Men’s Health*.

[32] Kume T., Calan M., Yilmaz O. (2016). A possible connection between tumor necrosis factor alpha and adropin levels in polycystic ovary syndrome. *Journal of Endocrinological Investigation*.

[33] Cho Y. H., Craig M. E., Donaghue K. C. (2014). Puberty as an accelerator for diabetes complications. *Pediatric Diabetes*.

[34] Gao S., McMillan R. P., Zhu Q., Lopaschuk G. D., Hulver M. W., Butler A. A. (2015). Therapeutic effects of adropin on glucose tolerance and substrate utilization in diet-induced obese mice with insulin resistance. *Molecular Metabolism*.

[35] Akcilar R., Kocak F. E., Simsek H. (2016). Antidiabetic and hypolipidemic effects of adropinin streoptozotocin-induced type 2 diabetic rats. *Bratislava Medical Journal*.

[36] Sawicka B., Bossowski A., Szalecki M. (2010). Relationship between metabolic parameters and thyroid hormones and the level of gastric peptides in children with autoimmune thyroid diseases. *Journal of Pediatric Endocrinology and Metabolism*.

